# Electrostatic Self-Assembled Synthesis of Amorphous/Crystalline g-C_3_N_4_ Homo-Junction for Efficient Photocatalytic H_2_ Production with Simultaneous Antibiotic Degradation

**DOI:** 10.3390/nano13222964

**Published:** 2023-11-17

**Authors:** Yilin Pan, Kai Qiao, Chuangyu Ning, Xin Wang, Zhiquan Liu, Zhihong Chen

**Affiliations:** 1 Key Laboratory for Water Quality and Conservation of the Pearl River Delta, Guangzhou University, Guangzhou 511370, China; 2 International Academy of Optoelectronics at Zhaoqing, South China Normal University, Liyuan Street, Zhaoqing 526238, Chinawangxin@scnu.edu.cn (X.W.); 3 Institute of Carbon Neutrality, Zhejiang Wanli University, Ningbo 315100, China; 4 National Center for International Research on Green Optoelectronics, South China Academy of Advanced Optoelectronics, South China Normal University, Guangzhou 510006, China

**Keywords:** H_2_ production from wastewater, carbon nitride, homo-junction

## Abstract

g-C_3_N_4_ has been regarded as a promising photocatalyst for photo-reforming antibiotics for H_2_ production but still suffers from its high charge recombination, which has been proven to be solvable by constructing a g-C_3_N_4_ homo-junction. However, those reported methods based on uncontrollable calcination for preparing a g-C_3_N_4_ homo-junction are difficult to reproduce. Herein, an amorphous/crystalline g-C_3_N_4_ homo-junction (ACN/CCN) was successfully synthesized via the electrostatic self-assembly attachment of negatively charged crystalline g-C_3_N_4_ nanorods (CCN) on positively charged amorphous g-C_3_N_4_ sheets (ACN). All the ACN/CCN samples displayed much higher photo-reforming of antibiotics for H_2_ production ability than that of pristine ACN and CCN. In particular, ACN/CCN-2 with the optimal ratio exhibited the best photocatalytic performance, with a H_2_ evolution rate of 162.5 μmol·g^−1^·h^−1^ and simultaneous consecutive ciprofloxacin (CIP) degradation under light irradiation for 4 h. The UV–vis diffuse reflectance spectra (DRS), photoluminescence (PL), and electrochemical results revealed that a homo-junction is formed in ACN/CCN due to the difference in the band arrangement of ACN and CCN, which effectively suppressed the charge recombination and then led to those above significantly enhanced photocatalytic activity. Moreover, H_2_ was generated from the water reduction reaction with a photogenerated electron (e^−^), and CIP was degraded via a photogenerated hole (h^+^). ACN/CCN exhibited adequate photostability and reusability for photocatalytic H_2_ production with simultaneous CIP degradation. This work provides a new idea for rationally designing and preparing homo-junction photocatalysts to achieve the dual purpose of chemical energy production and environmental treatment.

## 1. Introduction

Hydrogen (H_2_) production from photocatalytic water splitting has been regarded as one of the most promising strategies to convert solar energy into available chemical energy for addressing the increasing energy crisis [1]. In the past decades, the efficiency of the photocatalytic H_2_ production technology has rapidly increased as a result of the in-depth understanding of the behaviors of photo-carries and photocatalytic water splitting reactions and the application of sacrificial reagents as hole (h^+^) scavengers [2,3,4]. However, those reported sacrificial reagents, including alcohols [5], triethanolamine [6], lactic acid [7], etc., are valued energy sources, which will offset the economic value of the H_2_ generated from photocatalysis and limit its future industrial procedure. Thus, it is highly desirable to promote further industrial photocatalytic H_2_ production for novel system development.

Recently, an evolutionary photocatalytic H_2_ production with organic wastewater as the novel sacrificial reagent has been reported and received increasing attention for its realization of organic pollutants degradation with the simultaneous conversion of organic pollutants into H_2_ energy [8,9,10]. In the reported photocatalysts used in photo-reforming antibiotics for H_2_ production, g-C_3_N_4_ is currently one of the most attractive candidates due to its metal-free nature, non-toxicity, and stability against photo and chemical corrosion [11,12,13]. In 2017, Chen et al. first reported that g-C_3_N_4_ could be used as the photocatalyst for photo-reforming of antibiotics for H_2_ production, in which the poisonous macrolide antibiotics were degraded with simultaneous H_2_ gas production [14]. However, the photo-reforming of antibiotics for H_2_ production activity of pristine g-C_3_N_4_ is limited by its intrinsic drawbacks of narrow visible-light absorption and rapid recombination rate of photogenerated carriers [15].

The existing research has demonstrated that constructing hetero-junction is an effective route to enhance the photocatalytic activity of g-C_3_N_4_ by increasing light absorption and photogenerated carrier separation. For instance, Wang et al., found that constructing a Z-scheme Ag/g-C_3_N_4_-Ag-Ag_3_PO_4_ hetero-junction can effectively enhance the efficiency of photocatalytic degradation with the simultaneous H_2_ production of g-C_3_N_4_ [16]. Liu et al. developed a compact hetero-junction VS_2_@g-C_3_N_4_ with S vacancies, which showed an enhanced synergistic H_2_ production rate and wastewater degradation efficiency of 16.0 times that of pristine g-C_3_N_4_ [17]. Nevertheless, these g-C_3_N_4_-based hetero-junctions are mainly built from organic g-C_3_N_4_ and inorganic semiconductors, which are not only subjected to their energy band configurations but drastically affected by their surface/interface and crystal nature. Moreover, organic g-C_3_N_4_ is not easy to bond on the surface of inorganic semiconductors, which leads to high interface transmission resistance and thus facilitates the recombination of photo-generated charges at organic–inorganic interfaces.

Compared with hetero-junction, the homo-junction built from one substance with different crystal phases shows a similar identity on both sides of the interface, which could maintain continuous band bending and thus facilitate the carrier transfer across the interface more smoothly [18]. Notably, the electronic band structure of the g-C_3_N_4_ derived from different nitrogen-rich precursors is generally different, which makes it possible to construct a g-C_3_N_4_-based homo-junction. To date, various g-C_3_N_4_-based homo-junctions, including g-C_3_N_4_/S-g-C_3_N_4_ [19], morphology-dependent [20], triazine/heptazine [21], and amorphous/crystalline [22] g-C_3_N_4_ homo-junctions, have been developed, which exhibit a significantly enhanced photocatalytic activity for single organic pollutant degradation or H_2_ evolution from water splitting. However, there is scarce research on the application of g-C_3_N_4_-based homo-junction in photo-reforming. Moreover, the current g-C_3_N_4_-based homo-junction is usually fabricated via one-step or multi-step calcination of different raw nitrogen-rich materials [23,24,25]. Since the polymerization process of nitrogen-rich materials is easy to become out of control in calcination, interface defects and uncontrolled components would occur, thus making these g-C_3_N_4_-based homo-junctions difficult to reproduce. Therefore, more repeatable approaches for preparing g-C_3_N_4_-based homo-junction and their application, along with the mechanism for photo-reforming antibiotics for H_2_ production, are urgently required.

In the current work, for the first time, we developed a repeatable electrostatic self-assembly method for facile and precise preparation of amorphous/crystalline g-C_3_N_4_ homo-junction (ACN/CCN) and applied it to the photo-reforming of CIP for H_2_ production. As expected, the amorphous/crystalline g-C_3_N_4_ homo-junction showed much higher photocatalytic activity than that of amorphous and crystalline g-C_3_N_4_ under visible light, which resulted from the highly efficient charge transfer and separation.

## 2. Experimental Section

### 2.1. Materials

Urea, melamine, NaCl, ciprofloxacin, and Nafion were purchased from Shanghai Aladdin Bio-Chem Technology Co., Ltd. (Shanghai, China) and utilized without additional purification.

### 2.2. Synthesis of Amorphous and Crystalline g-C_3_N_4_ Homo-Junction

To prepare amorphous g-C_3_N_4_, 6 g of urea was put in the crucible and heated at 550 °C/min with a heating rate of 5 °C/min for 4 h. The attained product was gained and ground into powder, named ACN.

To prepare amorphous crystalline g-C_3_N_4_, 8 g of melamine was weighted in a crucible, heated with air at a rate of 12 °C/min to 500 °C for 4 h, then naturally cooled to room temperature and obtained as a white precursor. Then, 600 mg of the white precursor was mixed with 2.7 g of LiCl and 3.3 g of NaCl and ground and placed in a long-shape porcelain boat, which was heated to 550 °C in a tube furnace under an argon atmosphere at a rate of 12 °C/min and kept for 4 h. After naturally cooling down, the yellow powder was removed, dissolved in distilled water, and centrifuged several times. Finally, the product was dried in an oven at 60 °C, denoted as CCN.

The amorphous and crystalline g-C_3_N_4_ homo-junction (ACN/CCN) was fabricated via the electrostatic self-assembly method. A total of 0.1 g of ACN was ultrasonically dispersed in 100 mL of distilled water, whose pH was set to 4 by 10% of HCl, followed by 2 h of steady magnetic stirring to gain Dispersion A. The zeta potential of ACN in Dispersion A is 15.93 mV, which indicates that the surface of ACN is positively charged. Secondly, 10, 30, and 40 mg of CCN were ultrasonically dispersed in 50 mL of distilled water, whose pH was set to 4 by 10% HCl, and then swirled under constant magnetic stirring for 2 h to give Dispersion B. The zeta potential of CCN in Dispersion B is −30.29 mV, which indicates that the surface of CCN is negatively charged. Finally, Dispersion B was swiftly poured into Dispersion A and then stirred for 4 h. The opposite zeta potentials result in a strong electrostatic attraction between ACN and CCN; thus, a stable ACN/CCN homo-junction is obtained via the Coulomb electrostatic interaction. The mixing solution was centrifuged several times with distilled water and ethanol and then dried in an oven at 60 °C for 12 h. The sample was obtained and diagnosed as ACN/CCN-1, ACN/CCN-2, and ACN/CCN-3, respectively.

### 2.3. Material Characterization

The crystalline structure of ACN/CCN was characterized by an X-ray diffractometer (XRD, Bruker D8 ADVANCE, Bruker, MA, USA). The morphologies of the as-prepared samples were explored via scanning electron microscopy (ZEISS Ultra 55, ZEISS, Baden Wurttemberg, Germany) with EDS mapping and transmission electron microscopy. The ASAP 2460 Specific surface area tester from Micromeritics instrument Ltd. (Norcross, GA, USA) was used for Brunner-Emmet-Teller measurements (BET). X-ray photoelectron spectroscopy (XPS) was employed on the Thermo Scientific Nexsa G2 X-ray (Thermo Fisher Scientific, Waltham, MA, USA) Photoelectron Spectrometer. Fourier transform infrared spectroscopy was measured using the Nicolet™ iS™ 10 FTIR Spectrometer, Thermo Fisher Scientific Inc. (Waltham, MA, USA). The fluorescence spectrophotometric spectrum was analyzed using a Hitachi F-4600 (Hitachi Limited, Tokyo, Japan). The absorption ranges of the as-prepared samples were synthesized via diffuse reflectance spectroscopy (Hitachi U-41000 spectrophotometer, Hitachi Limited, Tokyo, Japan). The intermediate products from photocatalytic CIP degradation were evaluated using the 6460 triple-quadrupole LC/MS system from Agilent (Santa Clara, CA, USA).

### 2.4. Photoelectrochemical Analysis

The photoelectrochemical properties of the as-prepared samples were measured using an electrochemical analyzer (CHI660E, Shanghai Chenhua Co., Shanghai, China) in a standard three-electrode system. The procedure for the typical electrodes of the as-prepared sample is as follows: 15 mg of the as-prepared sample were ground thoroughly and then dropped with a 10 mL mixed solution (volume ratio of ethanol and Nafion is 95:5) and kept ground for 5 min to obtain a homogenous suspension. Then, a 2 mL suspension was dropped on the surface of the 1 × 1 cm^2^ SnO_2_:F (FTO) electrode. The working electrodes were obtained after heating at 150 °C for 2 h to evaporate the residual solvent. 0.1 M Na_2_SO_4_ serves as the electrolyte. The counter and reference electrodes were the Pt and saturated Ag/AgCl electrodes. A 300 W Xe lamp with an AM1.5 filter was utilized as a light source. EIS was recorded with a frequency range of 0.01 Hz to 100 KHz in light conditions at 0.2 V vs. the Ag/AgCl reference electrode. The photocurrent with ON/OFF cycles was measured at an applied potential of 0 V vs. Ag/AgCl. Mott–Schottky plots were obtained under direct current potential polarization at different frequencies (1000, 1500, and 2000 Hz) in dark conditions.

### 2.5. Photocatalytic Performance Analysis

The photocatalytic performances of the as-prepared sample have been evaluated via the photo-degradation of ciprofloxacin (CIP) simultaneously with H_2_ evolution using the Labsolar-6A online photocatalytic analysis system (Beijing Perfect Light Technology Co., Ltd., Beijing, China). Typically, a 50 mg catalyst was introduced into a quartz reactor with 100 mL of 500 ppm CIP solution containing 1 wt% Pt solution, which acts as the co-catalyst. Before illumination, the reactor was de-gassed to remove the dissolved air and oxygen. Then, Ar atmosphere was purged into the reactor to keep the pressure of the reaction system at air pressure. The 300 W Xenon lamp with an AM1.5 filter (Beijing Perfect Light Technology Co., Ltd., Beijing, China) was hired as the light resource. The gas chromatography using the Techcomp CG7900 (Techcomp, Beijing, China) investigated the amount of H_2_ for every hour, and the schematic of the photocatalytic H_2_ production measurement system is shown in Scheme S1. A 5 mL reaction solution has also been taken and separated via centrifugation to detect the degradation of CIP using a UV–vis spectrometer.

Before the subsequent renewed cycle of the recycle photo-degradation of ciprofloxacin (CIP) simultaneous with the H_2_ evolution experiment, about 48 mg of the photocatalyst can be collected via centrifugation and well cleaned with distilled water several times. If the cycle run of the photo-reforming of CIP for H_2_ production is conducted by directly using the remaining photocatalyst, H_2_ production and CIP degradation efficiency will inevitably be reduced. Therefore, each cycle run uses 50 mg of used ACN/CCN-2 as a photocatalyst, which was obtained with the previous cycle run. For example, to obtain 50 mg of used ACN for the 2nd run, the 1st run would need to be conducted twice. Due to the good stability of Pt loaded on ACN/CCN-2, it is only required to add Pt in the 1st run.

## 3. Results and Discussion

### 3.1. Morphology and Structure

**Figure 1:**
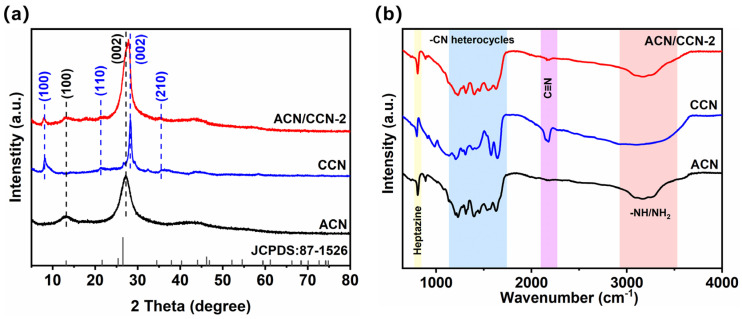
The (**a**) XRD pattern and (**b**) FTIR spectra for ACN, CCN, and ACN/CCN-2.

The crystal structure of the as-prepared ACN, CCN, and ACN/CCN samples was first obtained from the powder X-ray diffraction (XRD) patterns. As shown in Figure 1a, two identical diffraction peaks emerging at 2θ values of 13.1° and 27.5° were observed in the XRD pattern of ACN, which is indexed to the (100) and (002) diffraction planes of the typical amorphous heptazine-based g-C_3_N_4_ and consistent with the (100), (002) plane of g-C_3_N_4_ (JCPDS No. 87-1526) [26]. Additionally, a series of diffraction peaks appeared at the 2θ values of 8.2°, 22.1°, 28.5°, and 35.2° for CCN, which corresponded to the (100), (110), (002), and (210) planes of the crystal poly(heptazine imide) phase of g-C_3_N_4_ and were consistent with the other related work and theoretical calculated values [27,28,29,30]. Comparatively, the XRD pattern of ACN/CCN displayed a series of identical peaks at 2θ values of 8.2°, 13.1°, 22.1°, and 35.2° and a broad peak between 27.5° and 28.5° without other impure peaks, implying the successful synthesis of amorphous/crystalline g-C_3_N_4_ homo-junction using the electrostatic self-assembly method. Moreover, the broad peak between 27.5° and 28.5° reveals that the tight connection between amorphous and crystalline g-C_3_N_4_ in the homo-junction is critical for smooth interfacial charge transfer [31].

The FT-IR spectra were carried out to investigate the molecular structure and functional groups of the as-prepared samples. As shown in Figure 1b, ACN, CCN, and ACN/CCN possess the basic molecular and functional groups of the typical g-C_3_N_4_, which indicates that the basic main chemical structure of amorphous and crystalline g-C_3_N_4_ is not destroyed via the electrostatic self-assembly method. The obvious adsorption peak at 806 cm^−1^, series intense absorption bands in 1200–1700 cm^−1^, and broad adsorption peaks in 3000–3600 cm^−1^ corresponded to the ring-sextant out-of-plane bending vibration of s-triazine units and stretching vibration modes of C-N heterocycles and terminal amino groups [32]. Additionally, a distinct peak located at 2175 cm^−1^ corresponding to the terminal cyano groups (-C≡N) was found in the spectra of the CCN, which is caused by the catalytic pyrolysis of g-C_3_N_4_ in the presence of molten salt [33]. Notably, the distinct peak located at 2175 cm^−1^ can be found on the FT-IR spectra of ACN/CCN, which demonstrates the stability of CCN in the electrostatic self-assembly process and confirms the successful formation of homo-junction.

**Figure 2:**
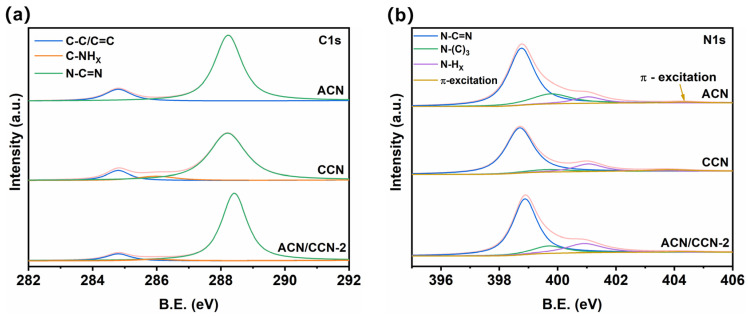
The high-resolution C1s (**a**), N1s (**b**), and XPS spectra of ACN, CCN, and ACN/CCN-2.

The XPS measurement was carried out to investigate the surface chemical states of the as-prepared photocatalysts and confirm the successful combination of ACN and CCN. The C1s high-resolution spectra (Figure 2a) have characteristic peaks at approximately 284.8, 285.6, and 288.4 eV that corresponded to the sp^2^ C-C/C=C, C≡N, and N-C=N bonds, respectively [34]. Obviously, CCN has a diffraction peak belonging to C≡N at about 286.5 eV. In the ACN/CCN-2 sample, the peak belonging to C≡N still exists, which is consistent with the results obtained via FT-IR and proves the successful combination of ACN and CCN. As for N1s’ high-resolution spectra (Figure 2b), four characteristic peaks at about 398.9, 399.75, 400.9, and 404.35 eV are attributed to the sp^2^-hybridized nitrogen (N-C=N), tertiary nitrogen (N-(C)_3_), N-H_x_, and π excitation, respectively [35]. Moreover, it is noteworthy that the N-C=N peak in the N1s and C1s XPS spectra of ACN/CCN-2 shows a weak positive shift compared to the ACN and CCN. It can be proven that electrons are transferred from ACN to CCN due to the excellent charge transfer capability at the phase interface, which is conducive to improving the photogenerated carrier migration rate [36].

**Figure 3:**
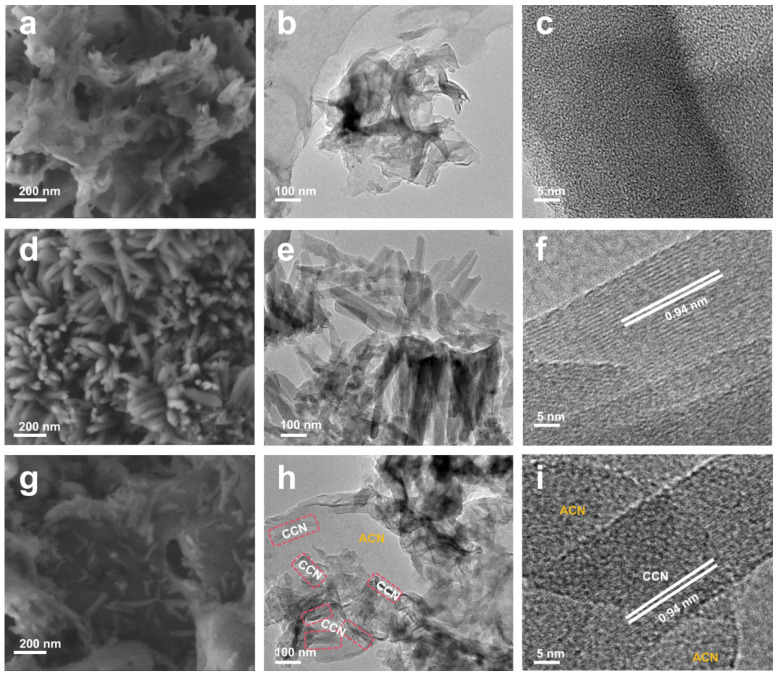
The SEM and TEM images of ACN (**a**–**c**), CCN (**d**–**f**), and ACN/CCN-2 (**g**–**i**).

Moreover, the crystalline morphology of the as-prepared ACN, CCN, and ACN/CCN were detected via SEM and TEM, and the results are shown in Figure 3. ACN features a typical wrinkle-like layer structure without any lattice fringe, which is inconsistent with the reported structure of amorphous g-C_3_N_4_ [37]. In the case of CCN, a nanorod morphology with a clear lattice fringe of 0.94 nm can be observed, strongly supporting the high-crystalline texture of CCN and in agreement with the XRD results. The nanorod size of CCN is about 200 nm in length and 30 nm in diameter. After electrostatic self-assembly, it can be clearly seen that CCN nanorods are stuck tightly on the surface of ACN nanosheets without obvious shape changes when forming the ACN/CCN homo-junction. Additionally, the HR-TEM of ACN/CCN reveals that the homo-junction between ACN and CCN is composed of amorphous sheets and nanorods with well-matched lattice fringes (100), indicating the successful construction of a tight amorphous/crystalline g-C_3_N_4_ homo-junction.

**Figure 4:**
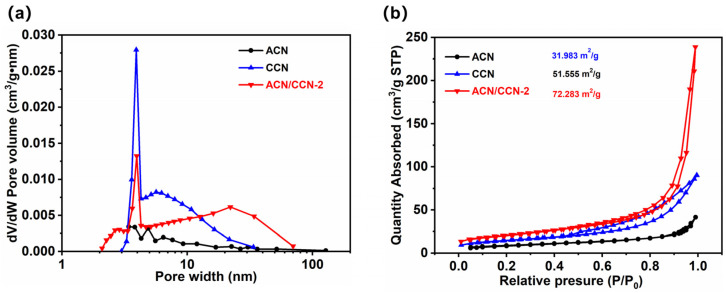
The pore size distribution (**a**) and N_2_ adsorption–desorption isothermal plots (**b**) of ACN, CCN, and ACN/CCN-2.

N_2_ gas adsorption–desorption isotherm and pore-size distribution curve analyses were performed to study the surface area and porosity of the as-prepared samples. ACN, CCN, and ACN/CCN present typical type IV N_2_ gas isotherm curves with an H1-type hysteresis loop, revealing the existence of mesoporous porosity of 2–50 nm (Figure 4). ACN shows a relatively low BET surface area of 31.983 m^2^/g because of the stacking of the layer structure, which makes it difficult to form holes, and the hole’s size is clustered below 8 nm. For the CCN sample, the crisscrossing of nanorods encourages the formation of more pores with sizes around 3–20 nm, which results in a higher BET surface area of 51.555 m^2^/g. Compared with ACN and CCN, the ACN/CCN-2 homo-junction displays an obviously higher BET surface area of 72.283 m^2^/g, which can provide abundant surface active sites for adsorption and surface reaction. As observed from the pores size distribution and SEM images, the enhanced surface area for ACN/CCN-2 is derived from the fact that the attachment of CCN nanorods on the surface of ACN can prop up the interlayer stack of ACN, which generates more pores centered at around 10–70 nm, which can provide active sites to enhance the photocatalytic performance of the photocatalyst.

**Scheme 1:**
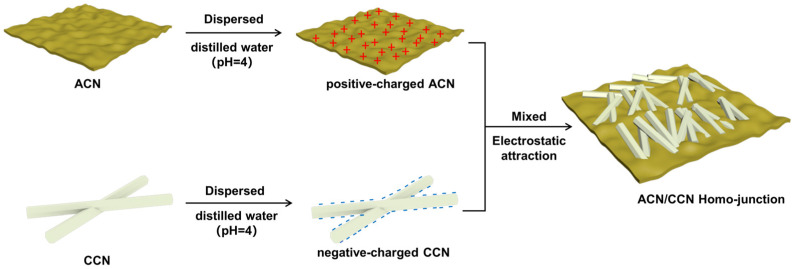
The schematic diagram of the preparation process of ACN/CCN homo-junction.

The above analysis proves that a homo-junction composed of amorphous g-C_3_N_4_ sheets and crystalline g-C_3_N_4_ nanorods is successfully synthesized via a facile self-assembly method (its schematic diagram is displayed in Scheme 1). After being dispersed in distilled water with a pH of 4, the surfaces of ACN and CCN show positive and negative charges, respectively. When they are mixed together, a strong Coulomb electrostatic attraction occurs between ACN and CCN, which binds them together to form a stable ACN/CCN homo-junction.

### 3.2. Photocatalytic H_2_ Evolution from Wastewater

**Figure 5:**
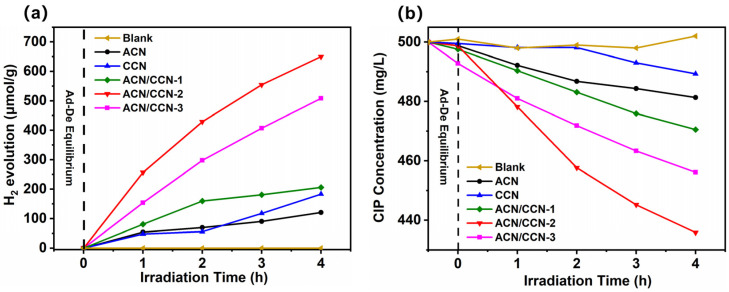
The photocatalytic H_2_ evolution (**a**) with simultaneous CIP degradation (**b**) of ACN, CCN, and ACN/CCN samples under simulated light irradiation.

The optimal dosage of co-catalyst Pt was evaluated by testing the H_2_ production at different dosages of co-catalyst Pt. As shown in Figure S1, there is a large increase in H_2_ production when the Pt dosage is boosted from 0.5 wt% to 1 wt%. However, as the Pt dosage was boosted to 1.5 wt%, the H_2_ production enhancement was not obvious. For practical application considerations, 1 wt% Pt was chosen as the optimal dosage. Figure 5a presents the photocatalytic H_2_ evolution performance activities of ACN, CCN, and ACN/CCN samples in CIP wastewater containing CIP with a concentration of 500 ppm degradation under visible light. Firstly, no H_2_ is detected without a photocatalyst, suggesting that light does not induce the water splitting reaction and cracking of CIP to generate H_2_. Both ACN and CCN display a very low photocatalytic H_2_ evolution from the CIP solution, which may be due to their high charge recombination and insufficient active sites. In sharp contrast, all ACN/CCN samples exhibit much higher photocatalytic H_2_ evolution from the CIP solution compared to ACN and CCN. In particular, ACN/CCN-2 exhibits the best performance in photocatalytic H_2_ evolution from CIP solution, with a H_2_ evolution rate of 162.5 μmol·g^−1^·h^−1^, which is about 5.42 and 3.56 times that of pristine ACN and CCN, respectively. In order to exclude the possibility that the difference in the H_2_ production activity of each catalyst is due to the different incorporation amounts of Pt, the amount of Pt incorporation in each photocatalyst after the reaction was measured via SEM-EDS. As shown in Figure S2, the doping amount of Pt after the reaction in each photocatalysts is consistent, so it can be proven that the difference in H_2_ production is due to the different migration rates of the photogenerated carriers of each photocatalyst. To reveal the positive effect of photocatalytic H_2_ evolution, the photocatalytic H_2_ evolution performance of ACN/CCN-2 in pure water was tested, and the result (Figure S3a) shows that the photocatalytic H_2_ evolution activity of ACN/CCN-2 is negligible in pure water, which suggests that the degradation process of CIP inducing by hole can be combined with photogenerated electron-induced water reduction for photocatalytic H_2_ evolution. Interestingly, CIP was gradually degraded as a function of irradiation time in the photocatalytic H_2_ evolution production process (Figure 5b), indicating that CIP not only can be degraded to achieve a globally concerned objective of environmental remediation but also can serve as a hole scavenger. In addition, compared with other g-C_3_N_4_-based photocatalysts (Table S1), ACN/CCN-2 is a promising photocatalyst for photo-reforming CIP for H_2_ production.

**Figure 6:**
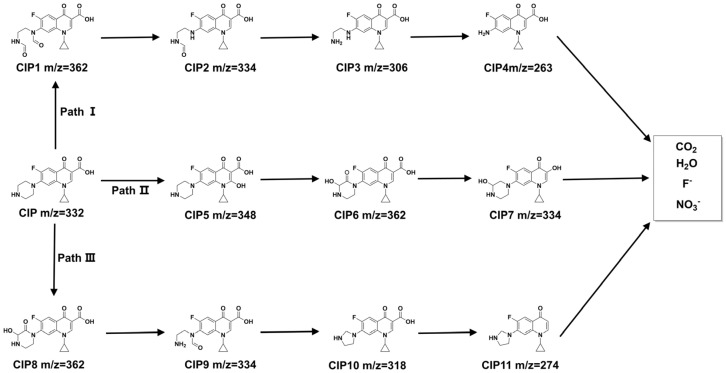
The degradation pathway of CIP in the present of ACN/CCN-2 homo-junction under simulated light irradiation.

Moreover, the change in the total organic content (TOC) of the CIP solution over time in the photocatalytic reaction and the results demonstrate that the TOC of the CIP solution decreases over irradiation time (Figure S3b). It is calculated that about 91.3% of the CIP is completely converted to CO_2_ and H_2_O, meaning that CIP is accurately removed along with H_2_ evolution under light irradiation. With the high-performance liquid chromatography-mass (HPLC-MS) analysis, possible photocatalytic degradation and removal pathways of CIP are displayed in Figure 6, and the HPLC-MS results are shown in Figure S4, which indicates that CIP is mineralized into CO_2_ and H_2_O via a series of indirect pathways. Judging from the TOC results and identified by-products from HPLC, the mineralization pathway of CIP, including three degradation pathways, is proposed and depicted in Figure 6. In pathway Ⅰ, the piperazinyl ring of CIP is oxidized to generate the dialdehyde derivative (CIP1), which then loses its aldehyde group to produce CIP2. CIP2 undergoes a decarboxylation reaction to form CIP3, whose amine, nitrogen, and formaldehyde are leaked to give CIP4 [38,39,40]. In pathway Ⅱ, CIP5 and CIP6 are formed from the continuous hydroxylation and carbonylation on the quinolone moiety and piperazinyl ring of CIP. With further degradation, the CIP6 product loses -CO to produce CIP7. In pathway Ⅲ, CIP8 is formed via piperazine ring oxidation in CIP. CIP8 undergoes -CO leakage and piperazinyl ring cleavage to generate keto-derivatives (CIP9), which further undergoes an intramolecular–bimolecular nucleophilic substitution reaction to form CIP10. The intermediate CIP11 could be immediately generated via the decarboxylation of the product CIP10 [41]. Finally, those intermediates are further mineralized into CO_2_ and H_2_O. The above results reveal that the photo-reforming of CIP for H_2_ production is established using amorphous/crystalline g-C_3_N_4_ homo-junction as a photocatalyst.

### 3.3. Photophysical Properties of the ACN/CCN Homo-Junction

**Figure 7:**
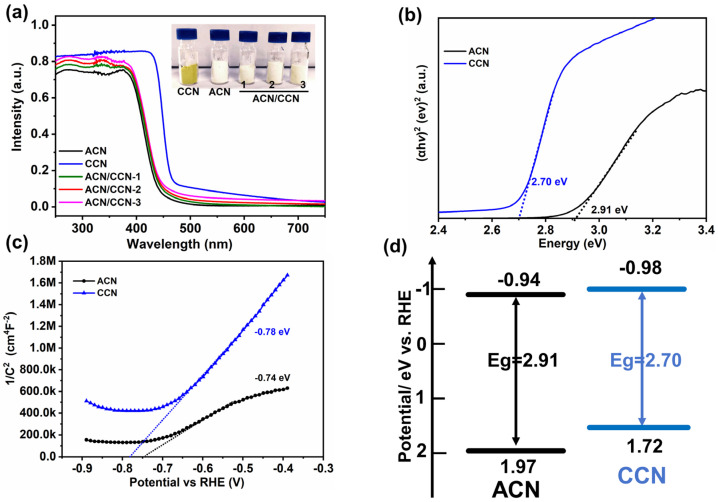
DRS spectra (**a**) of ACN, CCN, and ACN/CCN samples, Tauc plots (**b**), Mott–Schottky (**c**) plots, and band arrangement of ACN and CCN (**d**).

To investigate the reason why ACN/CCN possesses superior photocatalytic H_2_ evolution activity from wastewater and the connection between H_2_ evolution and pollutant degradation, systematical investigations relating to optical, band-structure, charge recombination, and transfer properties are reasonably detected. Firstly, the optical properties of ACN, CCN, and ACN/CCN were studied via the UV–vis diffuse reflection (DRS) method. According to the DRS results illustrated in Figure 7a, ACN shows an absorption edge of about 430 nm, which is a little smaller than that of typical stacked layer g-C_3_N_4_. The smaller absorption of ACN is due to the quantum effect of its stripped lamellar structure, which agrees with the SEM and TEM results [42]. On the other hand, CCN displays a higher absorption edge of 460 nm compared to the typical stacked layer g-C_3_N_4_, which is ascribed to the cyano group formed in the crystalline process, as confirmed with the above FT-IR results. The different bandgaps of ACN and CCN indicate that their band structures are different. For ACN/CCN samples, the absorption edge is between ACN and CCN, which shifts from ACN to CCN as the content of CCN increases, corresponding to the color change from pale yellow to deep yellow. These results reveal that the electrostatic self-assembly method effectively and precisely modifies the composition of the homo-junction.

To further confirm the band arrangement in the ACN/CCN homo-junction, the Tauc plots and Mott–Schottky plots of ACN and CCN were detected, and the results are displayed in Figure 7b,c. The bandgaps of ACN and CCN are calculated to be 2.91 eV and 2.70 eV from the Tauc plot based on the Kubelka–Munk function. The flat band potentials of ACN and CCN are evaluated as −0.74 and −0.78 eV vs. NHE from the Mott–Schottky plots, respectively. In addition, the positive slope of Mott–Schottky plots for ACN and CCN reveals that they are all n-type semiconductors, corresponding to their conduction band (CB) being about 0.2 eV higher than the flat band [43]. Therefore, the CB potentials of ACN and CCN are calculated to be −0.94 and −0.98 eV vs. NHE, respectively. Consequently, the valance band (VB) of ACN and CCN is estimated to be 1.97 and 1.72 eV vs. NHE based on the Eg and CB potentials, which confirms the formation of a homo-junction between ACN and CCN, whose band arrangement is depicted in Figure 7d.

**Figure 8:**
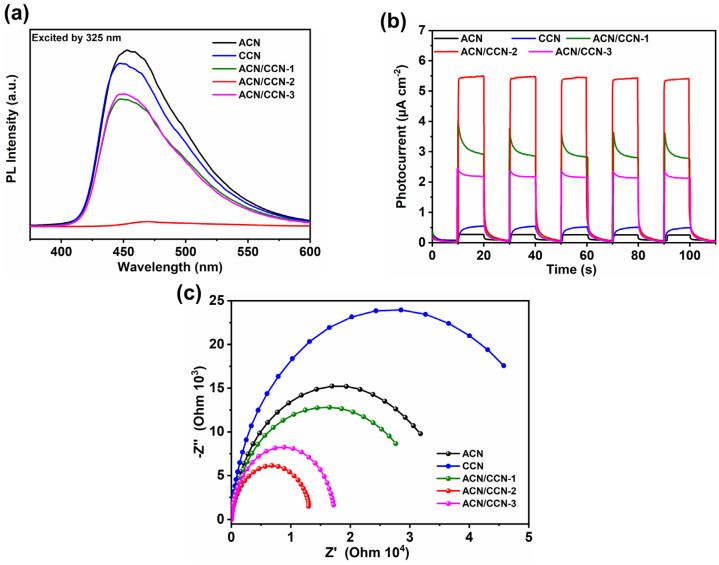
PL spectra (**a**), photocurrent (**b**), and electrochemical impedance spectroscopy (**c**) of ACN, CCN, and ACN/CCN samples.

Furthermore, the charge recombination and transfer properties of ACN, CCN, and ACN/CCN were explored in depth via photoluminescence (PL), electrochemical impedance spectroscopy, and photo-response current to uncover the homo-junction for superior photocatalytic H_2_ evolution with CIP degradation. As illustrated in Figure 8a, all samples display a broad PL peak with a range of 425–600 nm, which is consistent with the typical PL peak of g-C_3_N_4_. Obviously, the PL peak intensity of ACN/CCN samples is much lower than that of ACN and CCN, stating that the ACN/CCN homo-junction dramatically increases the successful electron transfer between ACN and CCN. Additionally, ACN/CCN-2 shows the lowest PL peak intensity in all ACN/CCN samples, which corresponds to the results of photocatalytic H_2_ evolution, further verifying the convenience of the electrostatic self-assembly method for adjusting the properties of homo-junction. Normally, the photocurrent and EIS Nyquist plots are sufficient evidence to reveal the photogenerated charge transfer property. Figure 8b displays the transient photocurrent responses of ACN, CCN, and ACN/CCN samples. The photocurrent intensities of ACN/CCN samples are all higher than those of ACN and CCN. As expected, ACN/CCN-2 features the highest photocurrent, pointing out that it has the highest separation rate and longest lifetime for photogenerated charges. In accordance with the PL and photocurrent results, EIS Nyquist plots (Figure 8c) also exhibit similar results, in which the ACN/CCN samples display a much smaller arc radius than ACN and CCN, and ACN/CCN-2 holds the smallest arc radius, indicating a smaller interface resistance for ACN/CCN.

### 3.4. Photocatalytic Mechanism of H_2_ Evolution from Wastewater

**Figure 9:**
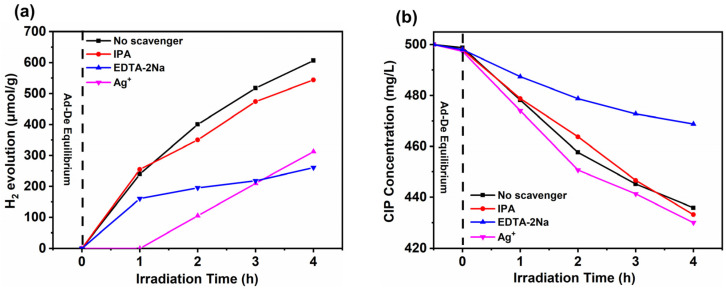
The photocatalytic H_2_ evolution (**a**) with simultaneous CIP degradation (**b**) in the presence of different trapping agents.

To evaluate the photocatalytic mechanisms of H_2_ production with simultaneous CIP degradation, the active species were probed in a trapping experiment. Figure 9 presents the photocatalytic vibration of H_2_ evolution with CIP degradation with the addition of different trapping agents. Ag^+^, IPA, and EDTA-2Na act as electron (e^−^), •OH, and hole (h^+^) trapping agents in this work, respectively. With the addition of Ag^+^, the H_2_ evolution rate drops to zero in the first hour, which suggests that the H_2_ is generated from the water reduction reaction with photogenerated electrons (Figure 9a). Then, the H_2_ evolution rate returns to the original rate of about 162.5 μmol·g^−1^·h^−1^, which is due to the depletion of Ag^+^, further confirming the source of H_2_. The photocatalytic H_2_ evolution rate shows a neglectful change in the presence of IPA, while a relatively obvious suppression of the H_2_ evolution rate occurs in the presence of EDTA-2Na, which results from the intermediate product after the reaction of EDTA-2Na is adsorbed on the surface of the photocatalyst and occupies the active sites [44]. As illustrated in Figure 9b, the CIP degradation rate was only slightly restrained with the addition of IPA but was strongly suppressed with the addition of EDTA-2Na, confirming that the degradation of CIP is induced by holes. Moreover, the H_2_ evolution rate drops to zero, and the CIP degradation rate did not decrease but slightly increased with the addition of Ag^+^, which demonstrates that the Ag^+^ electron extraction is faster than H^+^. This result also indicates that the limiting step of the system is H_2_ evolution.

**Scheme 2:**
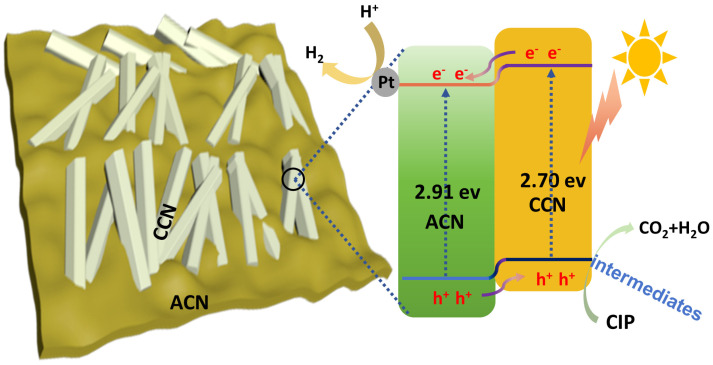
Proposed mechanism for the photocatalytic H_2_ evolution with CIP degradation in the presence of ACN/CCN.

Based on the band structure and trapping results, a mechanism for the photocatalytic H_2_ evolution with CIP degradation in the presence of ACN/CCN is proposed and illustrated in Scheme 2. Under simulated light irradiation, the ACN and CCN in the ACN/CCN homo-junction are excited and generate e^−^ on the CB and h^+^ on the VB. Subsequently, with the dragging force of the homo-junction, the e^−^ on the CB of CCN shifts to the CB of ACN, while the h^+^ on the VB of ACN transfers to the VB of CCN, which leads to rapid photogenerated charge separation. Finally, the e^−^ accumulated on the CB of ACN reduced water to generate H_2_, and the h^+^ accumulated on the VB of CCN degraded CIP, which achieved a dual objective of chemical energy production and environmental remediation.

### 3.5. Stability of ACN/CCN for Photocatalytic H_2_ Evolution from Wastewater

**Figure 10:**
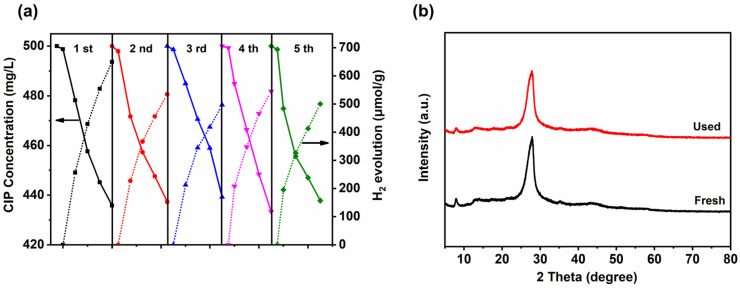
The five-cycle photocatalytic H_2_ evolution with CIP degradation performance (**a**) of ACN/CCN-2, and the corresponding XRD pattern before and after used (**b**).

The photostability and reusability of photocatalysts are essential for practical applications. Therefore, the stability of the ACN/CCN-2 homo-junction was judged using a five-cycle photocatalytic H_2_ evolution with CIP degradation in identical conditions. During the photoreaction process, Pt was attached to the photocatalyst via photodeposition. It was demonstrated via SEM-EDS (Figure S5) that the Pt content on the photocatalysts’ first run and second run remain stable and that the morphology of Pt can still be stabilized after the reaction, which proved that Pt had good stability during the cycling process. As shown in Figure 10a, no obvious decrease in photocatalytic H_2_ evolution and CIP degradation performance is observed for ACN/CCN-2 after five successive recycling photocatalytic reactions, demonstrating that the ACN/CCN has remarkable photostability and reusability for photo-reforming of CIP for H_2_ production. There is a slight decrease in H_2_ evolution in the second photocatalytic reaction, but then it will stabilize in the following cycle, which may be due to the adsorption of the intermediate products of the CIP on the surface of the photocatalyst, clogging the photocatalyst pores and covering the active sites. In addition, the ACN/CCN-2 after a five-cycle photocatalytic reaction shows a similar XRD pattern with the fresh ACN/CCN-2 (Figure 10b), which demonstrates the superior physical stability of the ACN/CCN homo-junction.

## 4. Conclusions

In summary, an amorphous/crystalline g-C_3_N_4_ composite with tunable properties was successfully fabricated via the electrostatic self-assembly method, in which negatively charged CCN is attached to the surface of positively charged ACN. Due to the difference in the band arrangement of ACN and CCN, a homo-junction structure is formed between ACN and CCN, which effectively suppresses the charge recombination, as confirmed with the quenched PL intensity, enhanced photocurrent, and smaller arc radius of the ACN/CCN homo-junction. Therefore, all ACN/CCN homo-junction samples showed higher photocatalytic activity in H_2_ evolution with CIP degradation than that of pristine ACN and CCN. Especially, ACN/CCN-2 exhibits the best performance in photocatalytic H_2_ evolution from CIP solution with a H_2_ evolution rate of 162.5 μmol·g^−1^·h^−1^ and a consecutive CIP degradation under light irradiation for 4 h. In this photocatalysis reaction, H_2_ was generated from the water reduction reaction by photogenerated e^−^, and CIP was degraded via photogenerated h^+^. Moreover, ACN/CCN exhibited adequate photostability and reusability for photo-reforming reactions. This work provides a simple route for rationally designing and synthesizing homo-junction to realize the dual purpose of chemical energy production and environmental treatment.

## Data Availability

Data are contained within the article.

## Supplementary Materials

The supporting information can be downloaded at https://www.mdpi.com/article/10.3390/nano13222964/s1. References [45,46,47,48,49,50,51,52] are cited in the supplementary materials.
