# Supplementary material for: Electrostatic Self-Assembled Synthesis of Amorphous/Crystalline g-C3N4 Homo-Junction for Efficient Photocatalytic H2 Production with Simultaneous Antibiotic Degradation
Source: Nanomaterials (Basel). 2023 Nov 17;13(22):2964. doi: 10.3390/nano13222964 (PMC10675752; doi:10.3390/nano13222964)
Supplement: Supplementary file 1 [file nanomaterials-13-02964-s001.zip › nanomaterials-2687576-supplementary.pdf]

Electrostatic Self-Assembled Synthesis of Amorphous/ Crystalline g-C_3N_4 Homo-Junction for Efficient Photocatalytic H_2 Production with Simultaneous Antibiotic Degradation

Yilin Pan [1], Kai Qiao [1], Chuangyu Ning [2], Xin Wang [2,3,4], Zhiquan Liu [1,*] and Zhihong Chen [1,*]

[1] Key Laboratory for Water Quality and Conservation of the Pearl River Delta, Guangzhou University, Guangzhou 511370, China
[2] International Academy of Optoelectronics at Zhaoqing, South China Normal University, Liyuan Street, Zhaoqing 526238, China; wangxin@scnu.edu.cn (X.W.)
[3] Institute of Carbon Neutrality, Zhejiang Wanli University, Ningbo 315100, China
[4] National Center for International Research on Green Optoelectronics, South China Academy of Advanced Optoelectronics, South China Normal University, Guangzhou 510006, China
* Correspondence: zhiquanliu@gzhu.edu.cn (Z.L.); chenzhihong@gzhu.edu.cn (Z.C.)

Table of contents

Table S1. Application of g-C_3N_4-based photocatalysts in photocatalytic H_2 production.

Scheme S1. Hydrogen measurement system schematic.

Figure S1. The ACN/CCN-2 photocatalytic H_2 production at different dosages of Pt.

Figure S2. The doping amount of Pt after the reacation of ACN (a), CCN(b) , ACN/CCN-2 (c).

Figure S3. (a) The photocatalytic H_2 evolution performance of ACN/CCN-2 in pure water and (b) TOC removal of CIP in the photocatalytic reaction.

Figure S4. The HPLC-MS identical intermediates of the degradation of CIP with ACN/CCN-2 under visible light irradiation.

Figure S5. The Pt conten on the ACN/CCN-2 1st run (a) and 2nd run (b).

Table S1. Application of g-C_3N_4-based photocatalysts in photocatalytic H_2 production.

Photocatalyst	Preparation	Light source	Solution Systems	Hydrogen evolution /pollutants degradation rate	Ref
GQDs/TCN	Calcination	300 W Xe (320 nm ≤ λ ≤ 780 nm)	10 ppm CIP	1μmol/g·h / 70%	[45]
C/N-TiO_2@C_3N_4NTs	Pyrolysis	300 W Xe (λ ≥ 420 nm)	20ppm RhB	9.67μmol/g·h / 70%	[46]
Ag/g-C_3N_4-Ag-Ag_3PO_4	Photoassisted isoelectric point	300 W Xe	10ppm LEV	55.41μmol/g·h / 90%	[16]
Pt@g-C_3N_4 nanorod	Calcination	300 W Xe (λ ≥ 420 nm)	10ppm 2,4-DNP	96μmol/g·h / 100%	[47]
CDs/CdS/gCN	Hydrothermal	300 W Xe (λ ≥ 420 nm)	10 ppm BPA	130μmol/g·h / 70%	[48]
2D BP/2D C_3N_4	Liquid-phase exfoliation	300 W Xe (λ > 400 nm)	10 ppm BPA	259μmol/g·h / 88%	[11]
g-C_3N_4 nanotubes	Hydrothermal-calcination	300 W Xe (λ > 400 nm)	10 ppm BPA	272.6μmol/g·h / 92%	[49]
g-C_3N_4 (oyster shell)	Single-step biomediated	125W Hg lamp （λ> 400 nm）	10 vol% TEOA	87 μmol/g·h	[50]
Re-CNN/CNN/Ox-CNN	Calcination	300W Xe（λ> 420 nm）	10 vol% TEOA	127.05μmol/g·h	[51]
UDCN-30	Calcination	150W Xe（λ> 420 nm）	10 vol% TEOA	190μmol/g·h	[18]
O-doped g-C_3N_4	One step template	300 W Xe（λ< 460 nm）	20 vol%TEOA	203.66 μmol/h	[52]
ACN/CCN	Electrostatic self-assembly	300 W Xe（AM1.5）	500 ppm CIP	162.5 μmol/h / 12.6%	This work

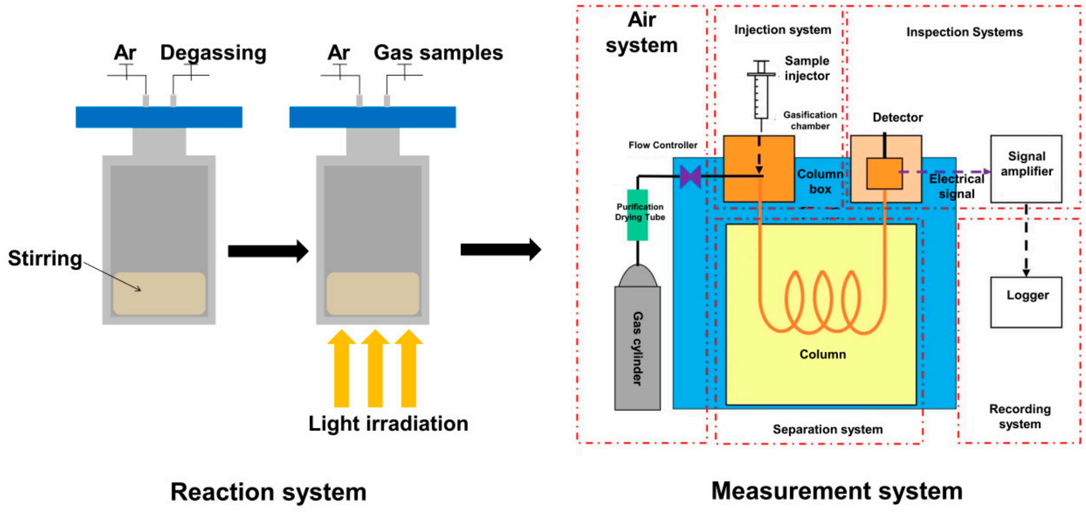

Scheme S1. Hydrogen measurement system schematic.

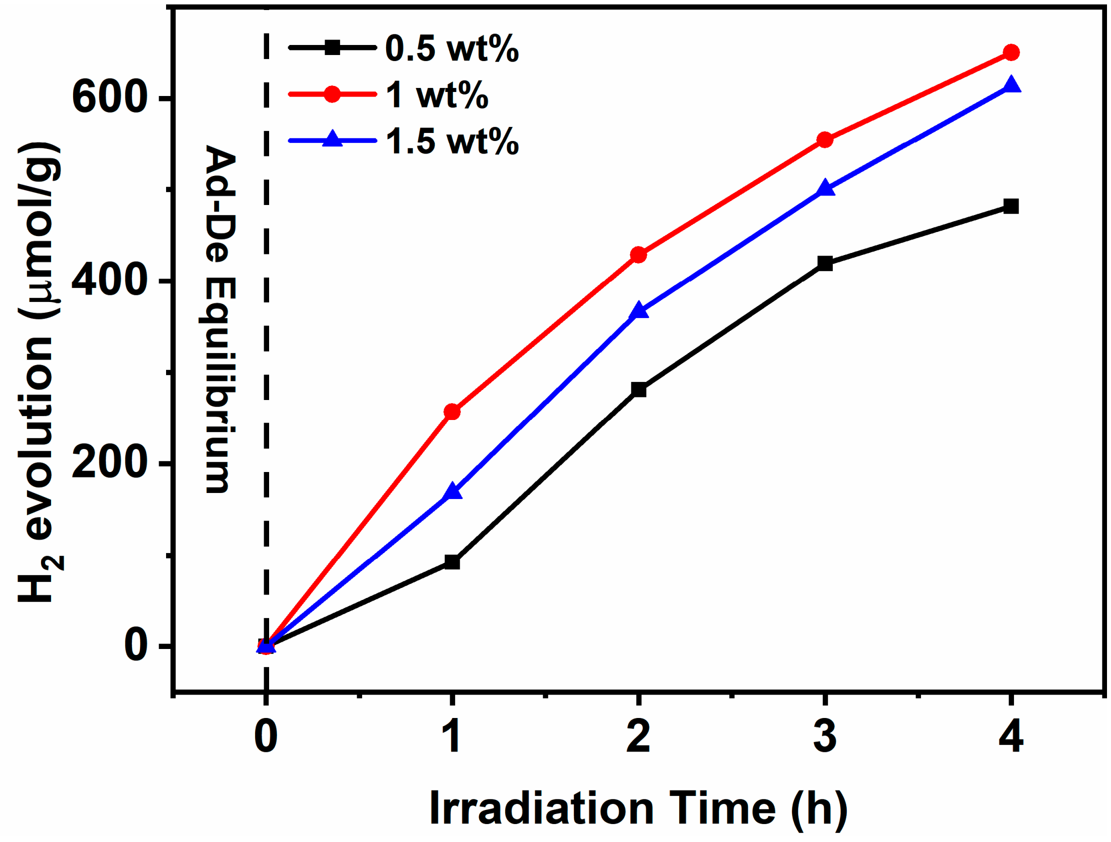

Figure S1. The ACN/CCN-2 photocatalytic H_2 production at different dosages of Pt.

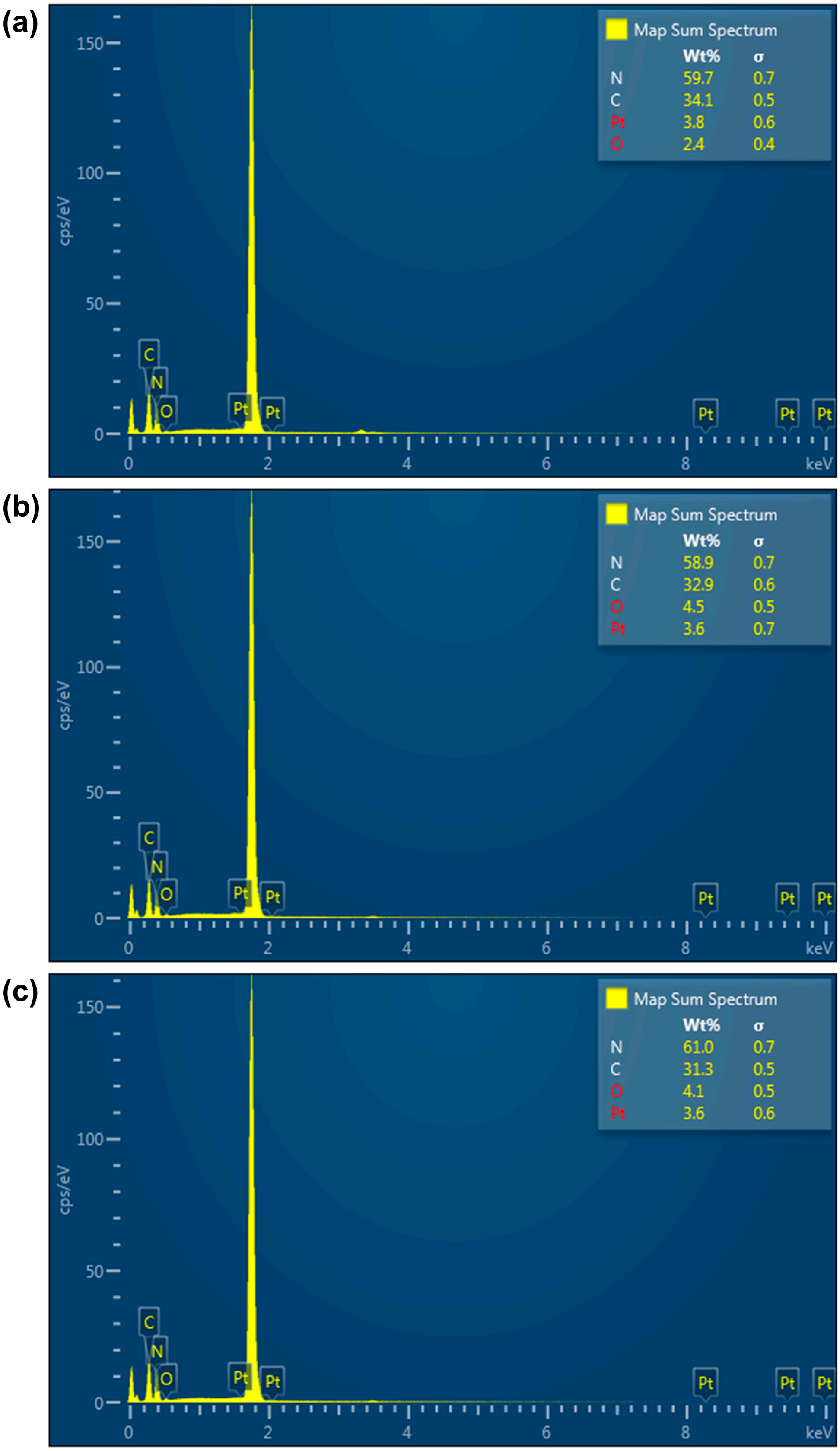

Figure S2. The doping amount of Pt after the reacation of ACN (a), CCN (b), ACN/CCN-2 (c).

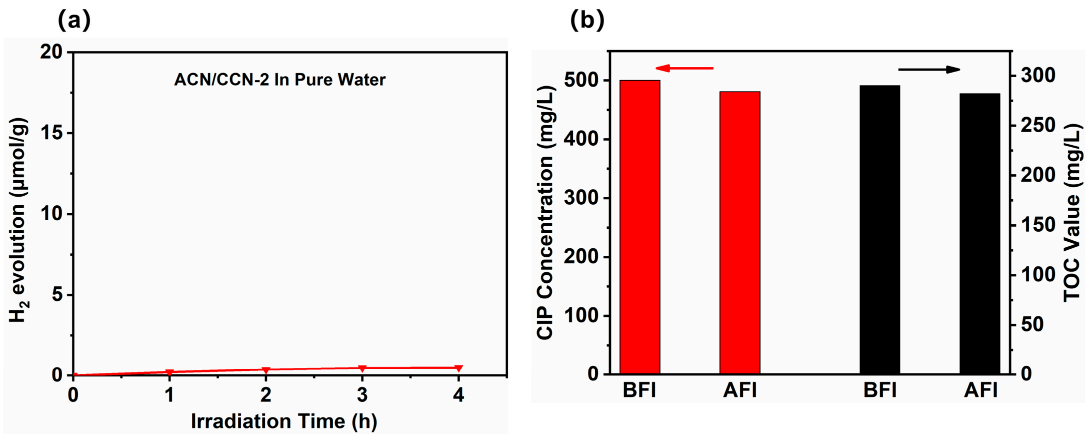

BFI = Before irradiation, AFI = After irradiation

Figure S3. (a) The photocatalytic H_2 evolution performance of ACN/CCN-2 in pure water and (b) TOC removal of CIP in the photocatalytic reaction.

Calculation of CO_2 selectivity

BCIP : CIP concentration before irradiation

ACIP : CIP concentration after irradiation

BTOC : TOC value before irradiation

ATOC : TOC value after irradiation

TTOC : Theoretical TOC value after irradiation = $\frac{ACIP \times BTOC}{BCIP}$

CO_2 selectivity = $\frac{BTOC - ATOC}{BTOC - TTOC} \times 100\%$

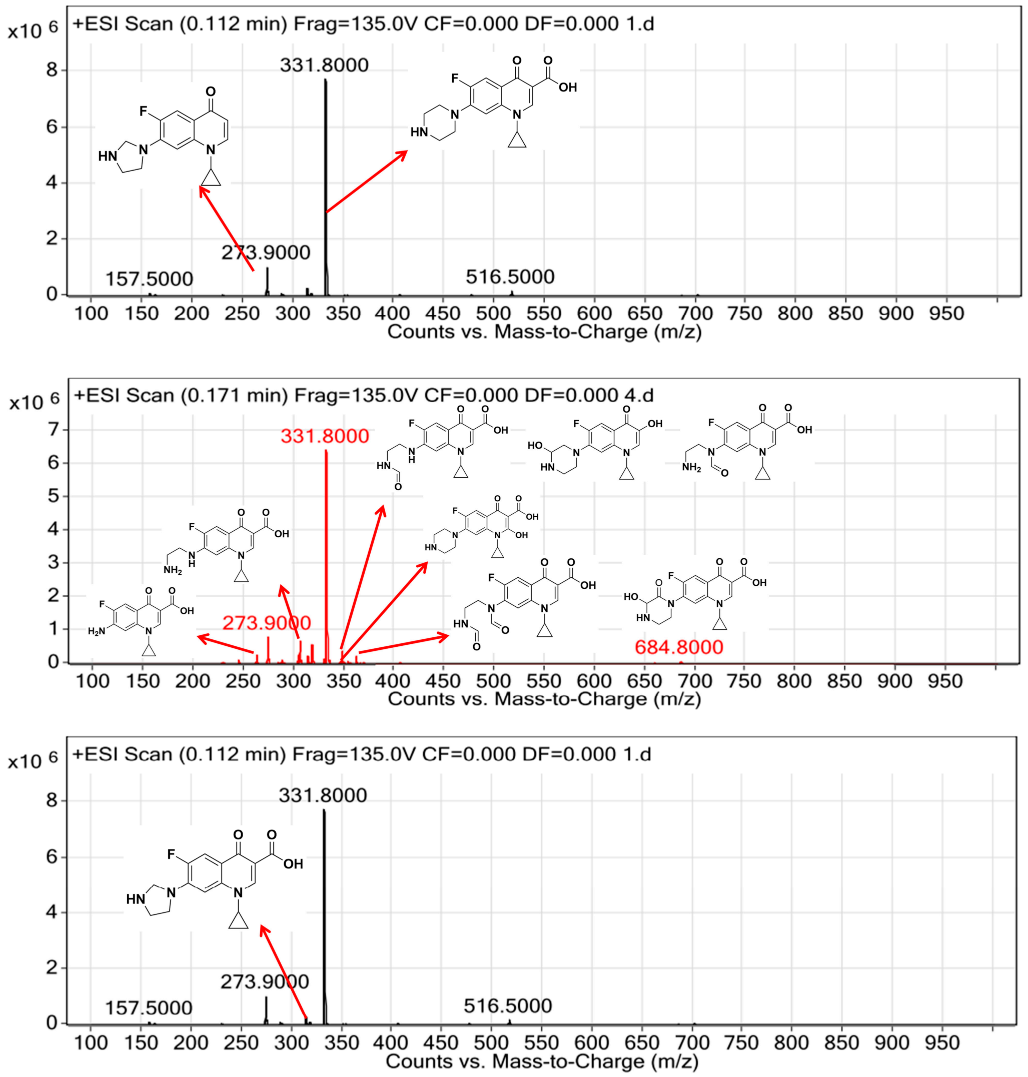

Figure S4. The HPLC-MS identical intermediates of the degradation of CIP with ACN/CCN-2 under visible light irradiation.

(a)

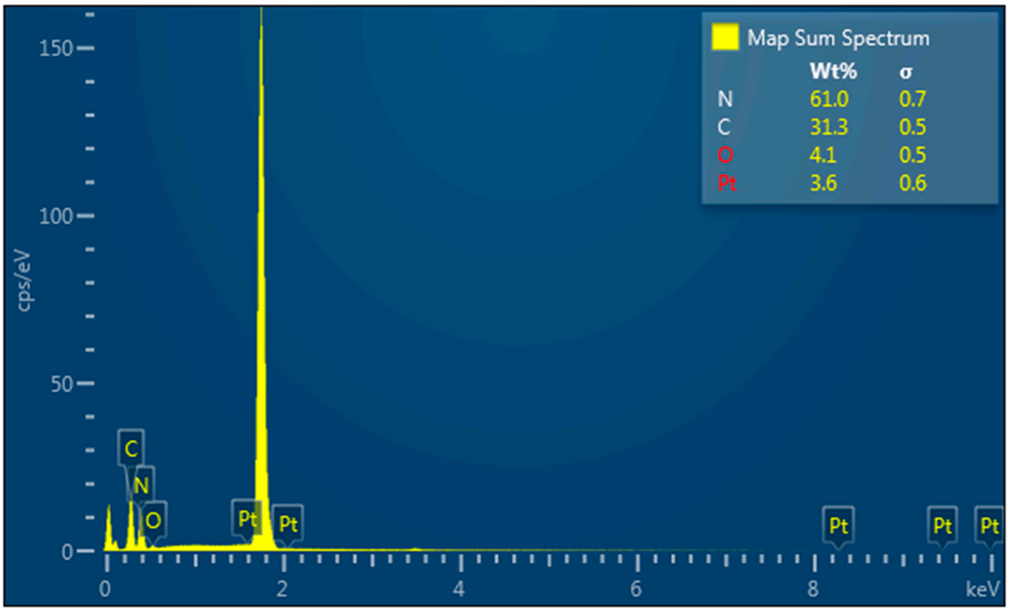

(b)

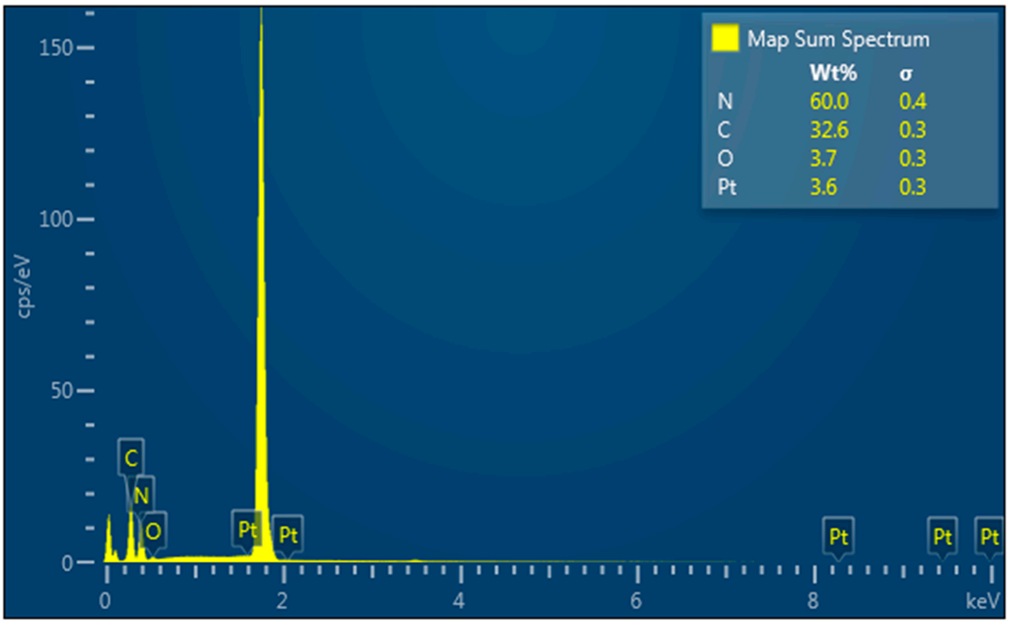

Figure S5. The Pt conten on the ACN/CCN-2 1st run (a) and 2nd run (b).
